# Facile Coating of Urea With Low-Dose ZnO Nanoparticles Promotes Wheat Performance and Enhances Zn Uptake Under Drought Stress

**DOI:** 10.3389/fpls.2020.00168

**Published:** 2020-02-26

**Authors:** Christian O. Dimkpa, Joshua Andrews, Job Fugice, Upendra Singh, Prem S. Bindraban, Wade H. Elmer, Jorge L. Gardea-Torresdey, Jason C. White

**Affiliations:** ^1^ International Fertilizer Development Center (IFDC), Muscle Shoals, AL, United States; ^2^ The Connecticut Agricultural Experiment Station, New Haven, CT, United States; ^3^ Department of Chemistry and Biochemistry, The University of Texas at El Paso, El Paso, TX, United States

**Keywords:** drought, micronutrients, nutrient delivery, crop performance, ZnO nanoparticle-coated urea, Zn nutrition

## Abstract

Zinc oxide nanoparticles (ZnO-NPs) hold promise as novel fertilizer nutrients for crops. However, their ultra-small size could hinder large-scale field application due to potential for drift, untimely dissolution or aggregation. In this study, urea was coated with ZnO-NPs (1%) or bulk ZnO (2%) and evaluated in wheat (*Triticum aestivum* L.) in a greenhouse, under drought (40% field moisture capacity; FMC) and non-drought (80% FMC) conditions, in comparison with urea not coated with ZnO (control), and urea with separate ZnO-NP (1%) or bulk ZnO (2%) amendment. Plants were exposed to ≤ 2.17 mg/kg ZnO-NPs and ≤ 4.34 mg/kg bulk-ZnO, indicating exposure to a higher rate of Zn from the bulk ZnO. ZnO-NPs and bulk-ZnO showed similar urea coating efficiencies of 74–75%. Drought significantly (p ≤ 0.05) increased time to panicle initiation, reduced grain yield, and inhibited uptake of Zn, nitrogen (N), and phosphorus (P). Under drought, ZnO-NPs significantly reduced average time to panicle initiation by 5 days, irrespective of coating, and relative to the control. In contrast, bulk ZnO did not affect time to panicle initiation. Compared to the control, grain yield increased significantly, 51 or 39%, with ZnO-NP-coated or uncoated urea. Yield increases from bulk-ZnO-coated or uncoated urea were insignificant, compared to both the control and the ZnO-NP treatments. Plant uptake of Zn increased by 24 or 8% with coated or uncoated ZnO-NPs; and by 78 or 10% with coated or uncoated bulk-ZnO. Under non-drought conditions, Zn treatment did not significantly reduce panicle initiation time, except with uncoated bulk-ZnO. Relative to the control, ZnO-NPs (irrespective of coating) significantly increased grain yield; and coated ZnO-NPs enhanced Zn uptake significantly. Zn fertilization did not significantly affect N and P uptake, regardless of particle size or coating. Collectively, these findings demonstrate that coating urea with ZnO-NPs enhances plant performance and Zn accumulation, thus potentiating field-scale deployment of nano-scale micronutrients. Notably, lower Zn inputs from ZnO-NPs enhanced crop productivity, comparable to higher inputs from bulk-ZnO. This highlights a key benefit of nanofertilizers: a reduction of nutrient inputs into agriculture without yield penalities.

## Introduction

Zinc oxide nanoparticles (ZnO-NPs; ≤100 nm in at least one dimension) are incorporated into a variety of industrial, medical, and household products to enhance quality and functionality (Piccinno et al., 2012). However, ZnO is a bioreactant, causing beneficial, sublethal, or toxic effects. Compared to bulk ZnO particles, such effects could be accentuated if exposure is to ZnO-NPs. This is as a result of the enhanced reactivity of nanoparticles arising from their small size and greater surface area, compared to bulk particles. Such heightened or nanoscale-specific effects have been observed in microbes, plants, and other terrestrial species (Dimkpa et al., 2012b; Dimkpa, 2014; Anderson et al., 2018; Rajput et al., 2018). In addition to greater nanoscale reactivity, the degree of the effects of ZnO-NPs also depends on dose, plant species and age, exposure route and duration, and environmental conditions such as pH and surface interactions with other soil components (Jośko and Oleszczuk, 2013; Watson et al., 2015; Mukherjee et al., 2016; García-Gómez et al., 2017; Dimkpa et al., 2019a). The contrasting (toxic *vs*. beneficial) effects of ZnO-NPs suggest they can be used as plant fertilizer if supplied at judicious doses. Accordingly, in the context of agriculture and human and environmental health, ZnO-NPs are being systematically assessed in plants for their enhanced ability to modulate productivity and nutrient use efficiency; confer tolerance to biotic and abiotic stresses; and fortify edible plant parts with Zn (Elmer and White, 2016; Raliya et al., 2016; Dimkpa et al., 2017a; Dimkpa et al., 2017b; Elmer et al., 2018; Dimkpa et al., 2018b; Zhang et al., 2018; Adisa et al., 2019; Dimkpa et al., 2019a; Dimkpa et al., 2019b).

One of the potential benefits of nanoscale fertilizers is the possibility of reducing nutrient application rates without sacrificing yield (Kottegoda et al., 2017), thereby saving on input costs and reducing the environmental footprint of chemical fertilizers in a sustainable manner. As previously discussed (Dimkpa and Bindraban, 2018), despite these benefits, the use as nanofertilizers of Zn and other essential microelements in large-scale field crop production appears currently unfeasible due to several potential complications. In the case of broadcast application of dry nanoparticles, the suspensibility of nanopowders in air would lead to large drift losses and potential human inhalation and subsequent health hazards for the handler and unintended biological targets. Whereas deep placement of the powder into the soil may mitigate handling hazards, particle adhesion to equipment surfaces, especially under wet conditions, could hamper efficient delivery. Similarly, suspensions of nanoparticles in water, especially of non-stabilized products (i.e., bare nanomaterials not surface-functionalized), for use as soil drench, foliar spray, or fertigation have at least two potential problems. In aqueous environments, the particles can dissolve into ions; or they can aggregate into non-nanoscale particles. Particle dissolution at a high rate obfuscates the effect of the nanofertilizer treatment owing to prevalent ionic activity (García-Gómez et al., 2017; Qiu and Smolders, 2017). In contrast to dissolution, aggregation of nanoparticles negates the definition of “nano” and associated size-specific reactivity. Thus, in both cases, particle transformation counteracts the underlying functionality of the nanofertilizer. Furthermore, the large difference in weight between urea granules and ZnO bulk powder causes particle size-dependent segregation when they are blended together and packaged for transportation. This problem of particle segregation will worsen when using ZnO-NPs for blending with urea. There is, therefore, a need to develop efficient and safe methods of delivering nano-scale nutrients to plants that also simultaneously streamline fertilizer application events in multi-nutrient fertilization regimes. One strategy that has been discussed in this regard is coating of finished bulk fertilizers such as urea or NPK granules with a nanopowder, to generate nano-enabled urea or NPK fertilizers (Dimkpa and Bindraban, 2018). Previous studies have described the coating of urea with ZnO-NPs and investigated the Zn dissolution kinetics from urea (Milani et al., 2012; Milani et al., 2015); notably, the effect of ZnO-NP-coated urea on plant performance was not evaluated.

Upon developing nano-enabled fertilizers for soil application, product efficacy in field crop production may be affected by natural events such as drought, which reduces nutrient mobility in soil, and consequently, uptake by plants. Indeed, drought continues to ravage different regions of the globe, with devastating consequences on soil nutrient bioavailability and crop productivity (Lesk et al., 2016; Moreno-Jiménez et al., 2019). Mechanistically, Zn can mitigate drought effects in crops (Karim et al., 2012; Dimkpa et al., 2017a; Dimkpa et al., 2019b), due to the role of Zn in metabolic processes regulating water dynamics. For example, under water stress, plants produce elevated amounts of abscisic acid (ABA) to optimize stomatal closure so as to conserve water (Karim and Rahman, 2015; Yang et al., 2018). Zn is known to increase ABA production in plants (Zengin, 2006; Yang et al., 2018), thereby enhancing stomatal regulation by ABA under water limitation.

Little is known as to whether coating of ZnO-NPs onto urea leads to better outcomes, in terms of performance and nutrient acquisition, for plants growing in challenged and unchallenged environmental conditions, or how this novel material would compare to other fertilization regimes such as with urea and ZnO-NPs applied separately. The objectives of the present study are to: i) understand differences in wheat performance using urea coated with ZnO-NPs *vs*. urea with separate ZnO-NP amendment; ii) determine whether ZnO-NPs coated on urea can mitigate the impact of drought stress on the performance of wheat; and iii) evaluate whether using a lower dose of ZnO-NPs can generate comparable effects as micro-scale (bulk) ZnO at a higher dose. Collectively, all effects were compared with those of bulk ZnO to determine the significance of nanoscale size.

## Materials and Methods

### Chemicals

Commercial ZnO-NPs (18 nm) was purchased from US Research Nanomaterials, Inc., Houston, Texas, USA. Bulk (≥1,000 nm) ZnO powder was purchased from Sigma-Aldrich, St Louis, Missouri, USA. For *in vitro* characterization, a suspension of the ZnO-NPs in water was probe-sonicated, then allowed to precipitate. The supernatant was pipette-filtered (20 µm pore) and diluted 1:1 in methanol. A drop (3 μl) of the suspension was mounted on a 300-mesh carbon-coated Cu grid. The nanoparticles were imaged using a transmission electron microscope (TEM; Hitachi 7800) in high resolution mode at an accelerating voltage of 80 kV. Furthermore, the solubility of both types of oxide particles in the experimental soil was evaluated by separately loading 10 mg of each powder in 20 g soil and incubating at room temperature for 24 h. The spiked soils were extracted in diethylenetriaminepentaacetic acid (DTPA) extracting solution (Lindsay and Norvell, 1978), shaken, filtered and then centrifuged at 10,000 rpm for 10 min. The supernatants were collected and analyzed for soluble Zn using inductively coupled plasma-optical emission spectroscopy (ICP-OES; model Spectro Arcos, SPECTRO Analytical Instruments GmbH, Kleve, Germany). The ZnO-NPs were not further characterized in soil for size-related properties. This is because of the complexity of soil medium, such as the presence of natural nano-size colloids, that would obfuscate the outcome of NP size characterization in soil.

### Facile Coating of Urea With ZnO Powder

Dry ZnO-NP (0.4 g) or bulk ZnO (0.8 g) powders were placed in transparent plastic bottles. To those were added 0.4 ml of commercial vegetable (canola) oil and 0.08 ml of black food color (McCormick, Hunt Valley, Maryland) for the nano-ZnO powder, or 0.8 ml vegetable oil and 0.16 ml of food color for the bulk ZnO powder. The urea granules and the ZnO powders are each white in color; thus, the food color provided a contrast that signaled the physical binding of the ZnO powders onto the urea surface that would otherwise be visually difficult to observe. The solutions were mixed to generate a grey-colored ZnO slurry. Urea granules were sieved in 2 mm cut-off sieves to obtain uniformly sized granules. Approximately 40 g of urea granules was added to the ZnO slurries. At these rates, the vegetable oil and food color amounted to 1.0 and 0.2% by weight, respectively, of the urea granule, for the control and nanopowder mixtures; and 2.0 and 0.4% of urea weight, respectively, for the bulk ZnO powder mixture. Similarly, the ZnO-NPs and the bulk ZnO corresponded to 1 and 2% by weight of the urea, respectively. The control urea was coated with vegetable oil and food coloring only and lacked addition of any ZnO powder. Each of the slurry-granular urea mixture was transferred to a low-speed mechanical shaker that generates roughly 32 rpm and all samples were allowed to mix overnight. The ZnO-coated urea was analyzed for the final Zn content by acid digestion (20 ml of 50% HCl), followed by boiling for 15 min, filtration, and dilution. ICP-OES was used to determine the Zn content. The original urea contained 46% N; after coating the N content was 45.7 and 45.3%, respectively, for the nano and bulk-coated products, indicating there was negligible change in the N content due to the coating process.

### Soil Preparation

The experimental soil is a sandy loam with the following characteristics: pH 6.87; organic matter content of 0.92%; bioavailable N and P of 4 and 2 mg/kg, respectively; and a bioavailable (DTPA-extractable) Zn of 0.1 mg/kg, indicating a Zn status well below the critical soil level for most crops, 0.5–1.0 mg/kg. The soil was amended with P (75 mg/kg; from monocalcium phosphate) and added into pots at 8 kg/pot, in three replicates. No K was added, as the soil contained more than sufficient amounts, at 1,903 mg/kg.

### Plant Growth

A greenhouse-based pot experiment involving winter wheat (*Triticum aestivum* var. Dyna-Gro 9522) was conducted in Muscle Shoals, Alabama (34.7448°N, 87.6675°W) during November-May of 2018–2019 (temperature, 1–33°C; relative humidity, 25–92%). Three wheat seeds were planted into the pots and were thinned to one seed upon germination. Two weeks after germination, the pots were fertilized with Zn-coated and uncoated urea; specifically, 217 mg of the urea was applied per kg of soil by sub-surface incorporation approximately 2 cm from the base of the plant and approximately 3 cm deep. Given 46% N, the amount of urea resulted in a nitrogen application rate of approximately 100 mg/kg (217 x 0.46). Coating of urea with 1 or 2% ZnO powder and applying 217 mg of the urea/kg soil resulted in the application of 2.17 mg ZnO/kg soil for nanoparticles, and 4.34 mg/kg soil for bulk particles. These levels of ZnO corresponded to ≈1.7 and ≈3.5 mg Zn/kg soil, respectively. Therefore, the respective Zn rates were directly used to amend in soil for the non-control uncoated urea treatments, corresponding to ≈17.4 mg ZnO-NPs, and ≈34.7 mg bulk ZnO powder per pot. Ultimately, five urea-Zn treatments were established, each in three replicates: i) control (urea coated with vegetable oil and food coloring); ii) control urea coated with ZnO-NPs (1%); iii) control urea + separate addition of ZnO-NPs (1%); iv) control urea coated with bulk ZnO (2%); and v) control urea + separate addition of bulk ZnO (2%). Each of these treatments was duplicated for the drought and non-drought conditions, resulting in a total of 10 treatments.

One week after Zn treatment, a portion of the plantlets were exposed to drought stress by maintaining the soil at 40% of field moisture capacity (FMC) until harvest. Forty % FMC was pre-determined using non-experimental potted soil. To this end, each pot was flooded with water until complete leaching for 24 h into a holder underneath. Pots were then weighed, and water in the soil at 100% FMC was determined by subtracting the weight of leached soil from the weight at 100% FMC. With plants in the pot, individual pots were weighed periodically to determine the required water per pot, since plant biomass varied per pot. The required amount of water per pot was then added to reach 40% FMC. This regime was maintained throughout the remainder of the plant growth period so as to keep the droughted plants at 40% FMC. In contrast, the non-stressed plants were kept at 80% FMC. During growth, time to flowering (panicle initiation by the primary shoot) was monitored; and upon full maturity, plants were harvested, grain yield was analyzed, and above-ground plants parts were analyzed for nutrient content.

### Plant Nutrient Analyses

Harvested plant tissues were oven-dried at 60°C until constant weight was achieved. Dried tissues were ground into powder using a Model 4 Thomas Wiley Laboratory Mill (Pennsylvania, USA). The ground tissues were acid-digested in a solution of 75% sulfuric acid (3 ml acid + 1 ml of 50% H_2_O_2_), heated for 1 h at 350°C, cooled to ambient temperature, and equilibrated with distilled H_2_O. Sub-samples of the prepared tissues were then subjected to Skalar segmented flow analysis for N and P, or to ICP-OES for Zn as noted above. Soil samples were also collected from the harvested pots for each treatment, to determine pH and bioavailable levels of N (as ammonium and nitrate fractions), P, and Zn. To this end, soil devoid of any root particles was collected for each treatment. The detailed procedures for the soil extraction and analyses of these elements have been described previously (Dimkpa et al., 2018a).

### Data Analysis

A two-way analysis of variance (ANOVA; OriginPro 2018) was used to determine significant differences in plant responses to the Zn treatments as a function of water status, for each variable, including vegetative and reproductive development, and nutrient content of plant and soil samples. A Fisher least significant difference (LSD) mean comparison was performed to further explore the differences with significant (p = 0.05) ANOVA. Considering that the actual Zn exposure rates among the ZnO treatments are different, the obtained values for the different plant measurement variables with significant ANOVA for Zn treatment (namely, time to panicle initiation, grain yield, and Zn uptake) were normalized by dividing the values by the respective exposure Zn rate.

## Results

### Characterization of ZnO Nanoparticles

**Figure 1:**
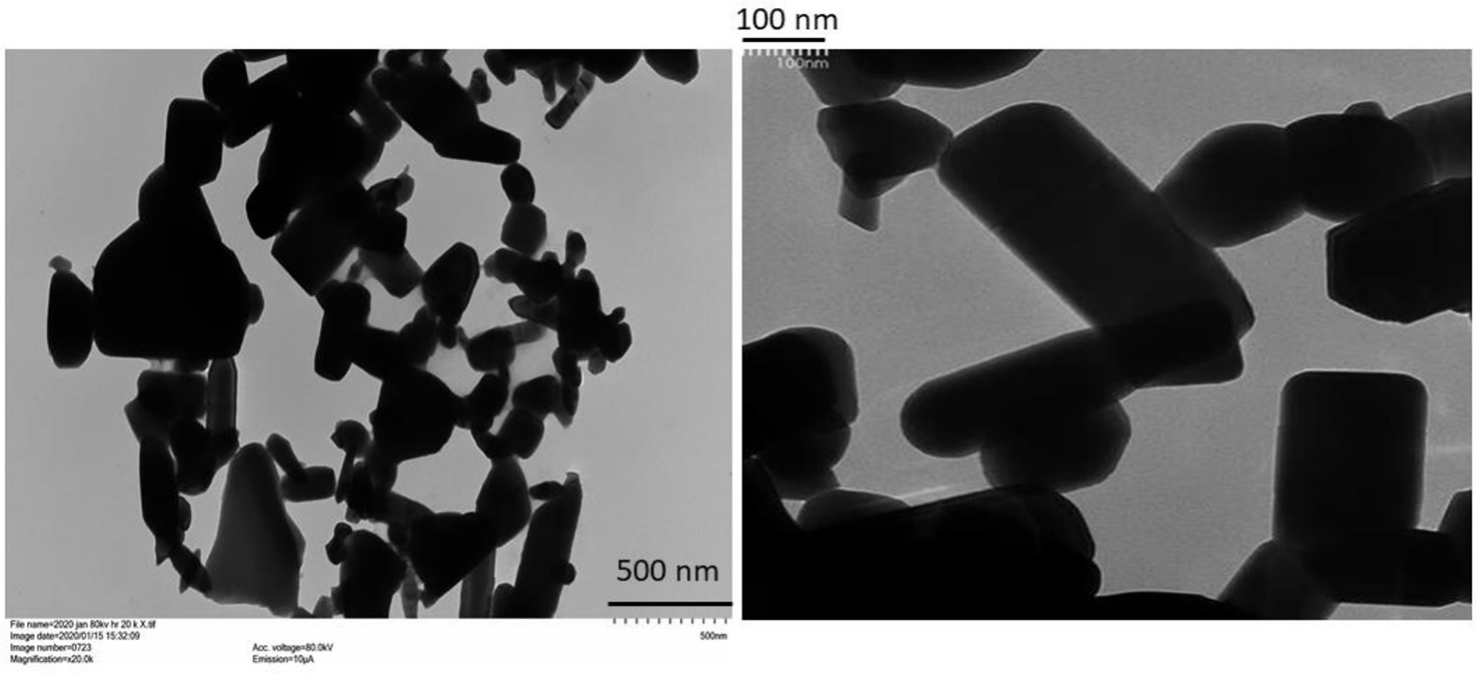
Transmission electron microscopy images of the ZnO-nanoparticles (NPs) used in the study (left: 500 nm resolution; right: 100 nm resolution). For the 500 nm image, each little vertical scale mark on lower right-hand side represents a 50-nm increment. For the 100 nm image, each little scale mark on upper left-hand side represents a 10-nm increment.

Images of the ZnO-NPs obtained by TEM are presented in **Figure 1**. The ZnO-NPs were present in multiple shapes, including rectangular, tubular, angular, and somewhat circular shapes. However, particles with amorphous shapes could also be seen. Particles with dimensions of less than 100 nm and others with dimensions greater than 100 nm were present, confirming the presence of both discrete nanoscale and aggregated structures. Solubility in the soil of the particles after 24 h was similar between the ZnO-NPs and bulk ZnO, with recoveries around 100% when 10 mg of ZnO was applied in 20 g of soil without plants.

### Facile Coating of Urea With ZnO Nano and Bulk Particles

**Figure 2:**
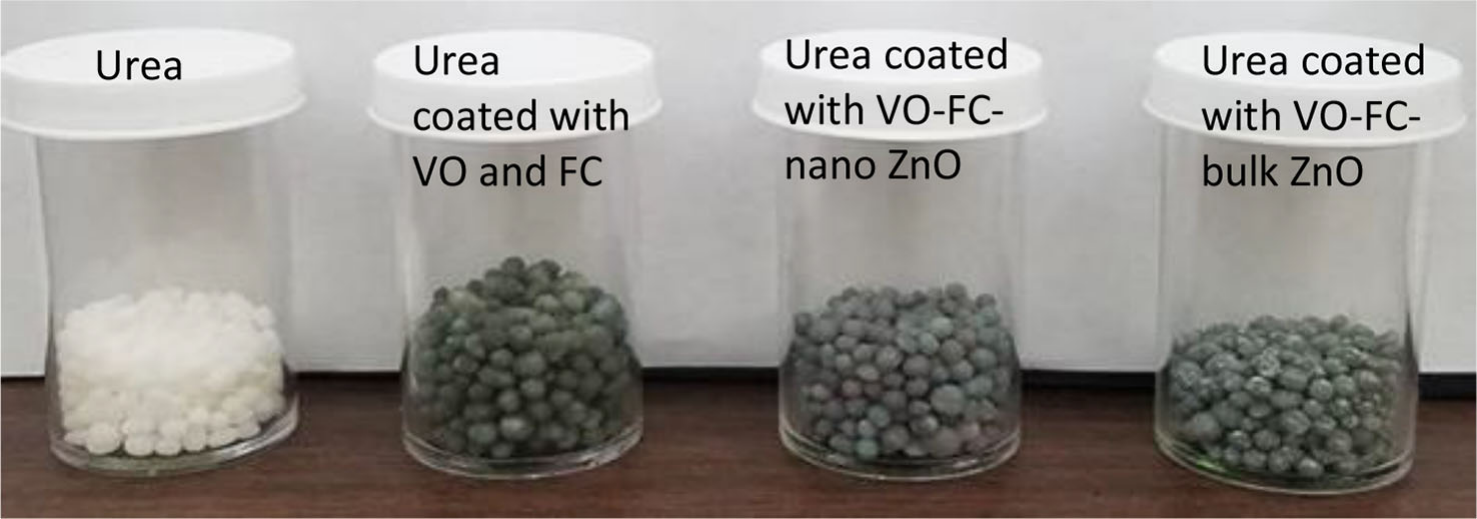
Urea granules, and urea granules coated with vegetable oil (VO) and food coloring (FC), without and with, ZnO nano or bulk powders.

Coating of urea with the nano-ZnO and bulk ZnO powders in a vegetable oil and food color slurry resulted in urea granules that were dark grey in color, in contrast to uncoated urea which is white. Visual observation showed that the entire surface of each urea granule became coated with the slurry, indicating uniform coverage (**Figure 2**). However, post-coating evaluation of the procedure indicated that not all of the slurry coated onto the urea; some dry particles with grey color stuck to the walls of the plastic container used for mixing the slurry and urea. Accordingly, the Zn content of the Zn-coated urea granules was determined, which showed that Zn in the nano-coated urea was only 0.74 ± 0.002% by weight of urea, contrary to the initial 1% target. Similarly, Zn in the bulk-coated urea was 1.51 ± 0.003% by weight of urea, as opposed to the 2% target. Given initial ZnO amounts of 0.4 and 0.8 g, this finding implies that only ≈0.3 g of the nano and ≈0.6 g of the bulk ZnO powder were eventually coated onto the 40 g urea. This suggested similar, 74 and 75%, urea coating efficiencies for the nano and bulk ZnO powders. From the point of view of plant exposure, this suggests that plants treated with the coated urea were exposed to slightly lower Zn rates of 1.6 and 3.3 mg/kg respectively, for the nanoscale and bulk oxide treatment, instead of the pre-targeted Zn rates of 1.7 and 3.5 mg/kg soil that plants treated with uncoated urea were exposed to (**Table 1**).

**Table 1:** Targeted and actual rates of added Zn exposure to wheat in soil. The term uncoated is with respect to Zn.

Trt/rate (mg/kg)	Control	Nano coated	Nano uncoated	Bulk coated	Bulk uncoated
Targeted Zn rate	0	1.7	1.7	3.5	3.5
Actual Zn rate	0	1.6	1.7	3.3	3.5

### Effect of Zn-Coated Urea on the Development of Panicle in Wheat Under Drought Stress

**Figure 3:**
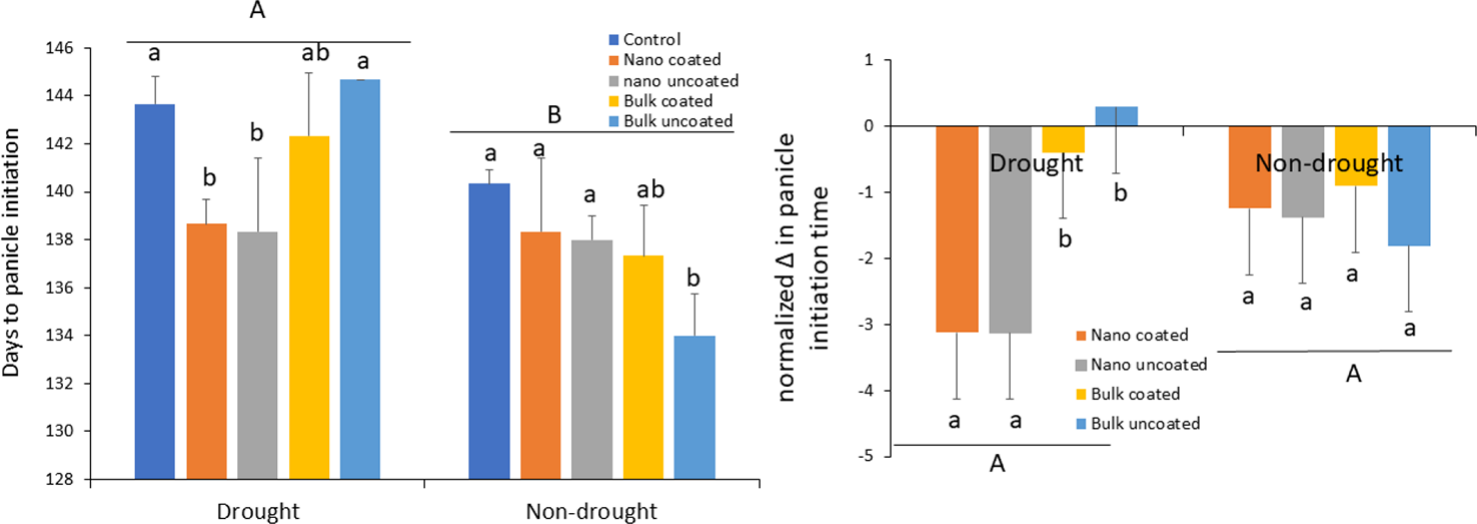
Left panel: effect of urea coated with ZnO-nanoparticles (NPs) or bulk ZnO and of separate ZnO-NP or bulk ZnO amendment with urea on the time to panicle development in wheat under drought and non-drought growth conditions. Right panel: change in panicle initiation time due to ZnO-NPs and bulk ZnO treatments normalized per unit (mg) of Zn. Values are means and standard deviations. Different uppercase letters above horizontal lines represent significant difference between the drought and non-drought condition. Different lowercase letters on bars indicate significant differences among the Zn treatments for each growth condition (n = 3). The term uncoated is with respect to Zn.

Compared to the adequate (80% FMC) water condition, drought (40% FMC) significantly delayed the time to panicle initiation in the wheat plants by 4–11 days, depending on the treatment (**Figure 3**; left panel). Under drought, ZnO-NPs strongly alleviated the delay in panicle initiation time, irrespective of whether the metal was coated onto urea or not. In contrast, bulk ZnO irrespective of coating did not affect the time to panicle formation under drought conditions. Overall, Zn fertilization had less significant effect on time to panicle initiation under the non-drought scenario, although there was a clear trend for reducing the time to panicle formation in all Zn treatments. Only in the case of uncoated bulk ZnO was time to panicle formation significantly different from the control (**Figure 3**; left panel). However, when the effects of the actual exposure Zn rates were considered by expressing the values per mg Zn in terms of change in panicle time initiation and normalizing per unit Zn exposure, it can be seen that ZnO-NPs, regardless of coating or not onto urea, specifically facilitated plant development under drought, relative to the bulk ZnO treatments (**Figure 3**; right panel). However, as suggested by data in the left panel, this nano-specific effect was not apparent under the non-drought condition.

### Effect of Zn-Coated Urea on Wheat Grain Yield Under Drought Stress

**Figure 4:**
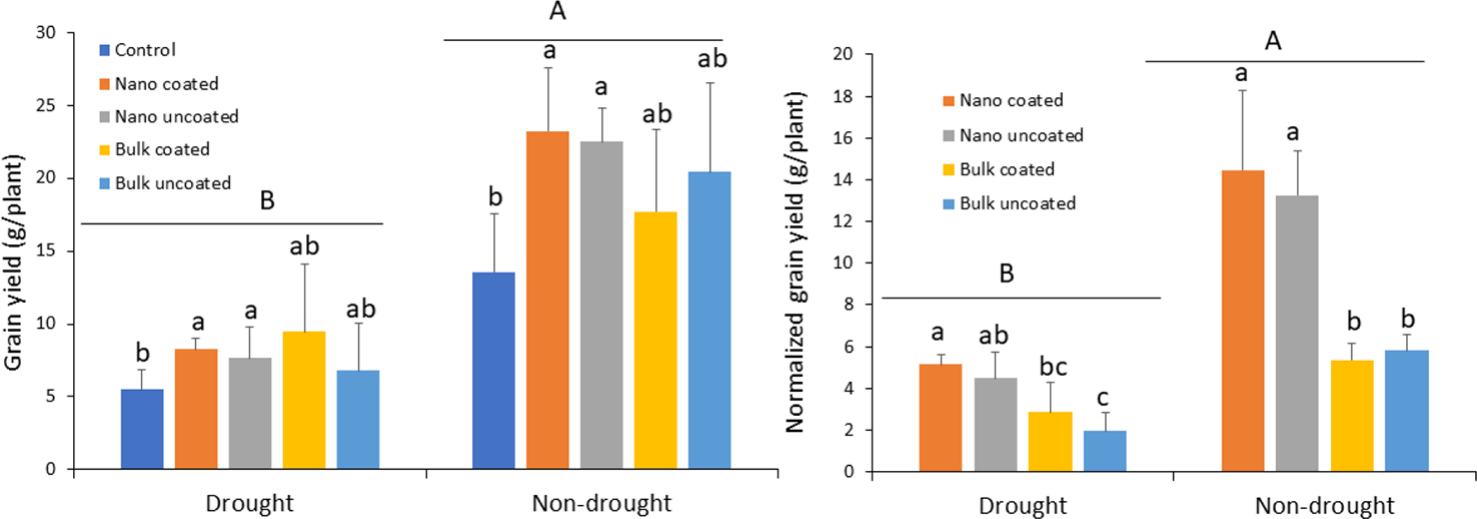
Left panel: effect of urea coated with ZnO-nanoparticles (NPs) or bulk ZnO and of separate ZnO-NP or bulk ZnO amendment with urea on the grain yield of wheat under drought and non-drought growth conditions. Right panel: normalized [per unit (mg) of Zn] values for the effect of ZnO-NPs and bulk ZnO on grain yield. Values are means and standard deviations. Different uppercase letters above horizontal lines represent significant difference between the drought and non-drought condition. Different lowercase letters on bars indicate significant differences among the Zn treatments for each growth condition (n = 3). The term uncoated in the legend is with respect to Zn.

Drought strongly reduced grain yield, relative to the non-drought condition, 59–73%, depending on the treatment. Under drought, ZnO-NPs significantly increased grain yield compared to the control and irrespective of whether coated onto urea or not. In contrast, bulk ZnO resulted in intermediate, non-significant effects on grain yield, relative to both the control and ZnO-NPs treatments. As with the nanoscale treatment, coating of urea with bulk ZnO did not produce a significantly different effect, compared to separate application of bulk ZnO (**Figure 4**; left panel). The effect of Zn on grain yield in the non-drought treatments mimicked that with the drought treatments. Unfortunately, the larger variation in the replicates (as indicated by the large error bars) of the bulk oxide treatments under both growth conditions, more so under non-drought, resulted in non-significant effects of that treatment, relative to the control (**Figure 4**; left panel). When the Zn effect was evaluated by normalizing in terms of per unit Zn, coating with Zn generated similar effect as not coating on grain yield, for both ZnO-NPs and bulk ZnO in the drought condition. However, the effect of nanoscale was apparent. Similar to the drought condition, the effect of nanoscale on yield was also clearly demonstrated, independent of Zn coating (**Figure 4**; right panel).

### Effect of Zn-Coated Urea on Zinc Uptake in Wheat Under Drought Stress

**Figure 5:**
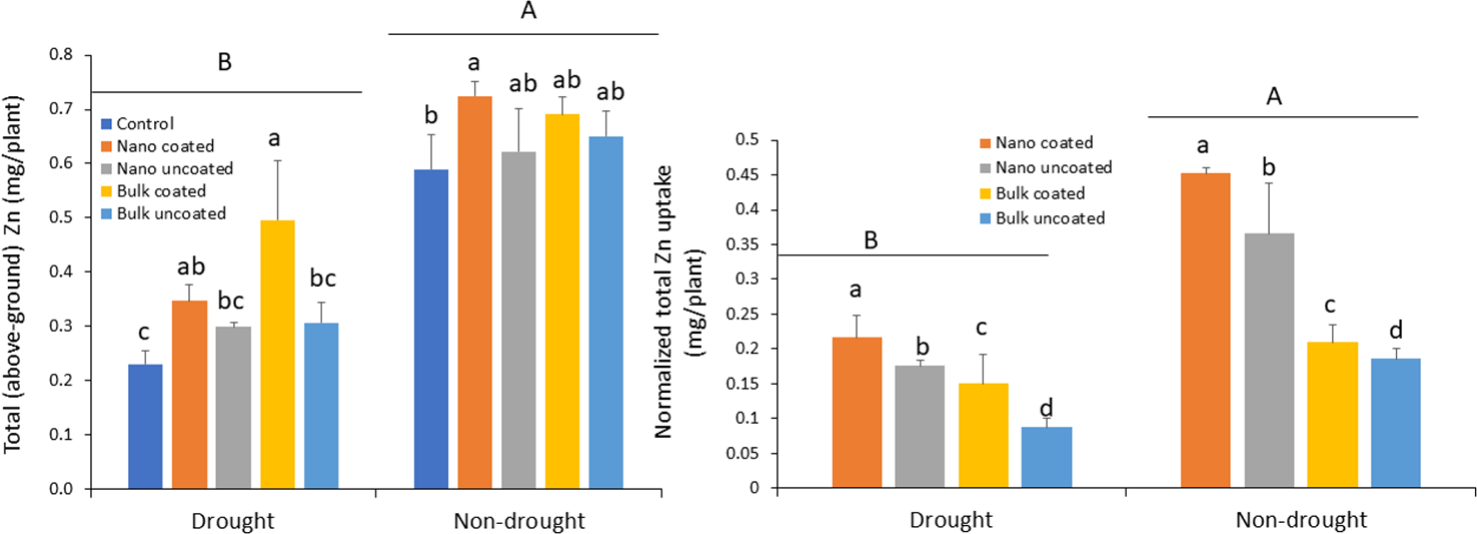
Left panel: effect of urea coated with ZnO-nanoparticles (NPs) or bulk ZnO and of separate ZnO-NP or bulk ZnO amendment with urea on above-ground accumulation of Zn in wheat under drought and non-drought growth conditions. Right panel: normalized [per unit (mg) of Zn] values for the effect of ZnO-NPs and bulk ZnO on Zn accumulation. Values are means and standard deviations. Different uppercase letters above horizontal lines represent significant difference between the drought and non-drought condition. Different lowercase letters on bars indicate significant differences among the Zn treatments for each growth condition (n = 3). The term uncoated in the legend is with respect to Zn.

Drought strongly reduced Zn uptake into above-ground wheat tissues relative to the non-drought condition, 29–116%, depending on the treatment. Under drought condition, coating of nanoscale or bulk ZnO particles onto urea significantly increased Zn uptake, relative to the control urea treatment. The effect of coating with nanoscale oxide particles was not significantly different from that of separate amendment of the nanoparticles. In contrast, urea coated with bulk oxide yielded significantly greater Zn uptake, compared to urea with the separately added bulk oxide (**Figure 5**; left panel). Under non-drought condition, only the ZnO-NP-coated urea significantly increased Zn uptake relative to the control; other treatments resulted in median values on Zn uptake when compared to the control and the ZnO-NP-coated urea (**Figure 5**; left panel). The normalized data confirmed coating of urea with ZnO-NPs to be more effective for Zn accumulation, relative to separate amendments. Similarly, ZnO-NPs were more effective for above-ground tissue Zn delivery than was bulk ZnO Zn type. These effects were consistent in both the drought and non-drought growth conditions (**Figure 5**; right panel).

### Effect of Zn-Coated Urea on Nitrogen and Phosphorus Uptake in Wheat Under Drought Stress

**Figure 6:**
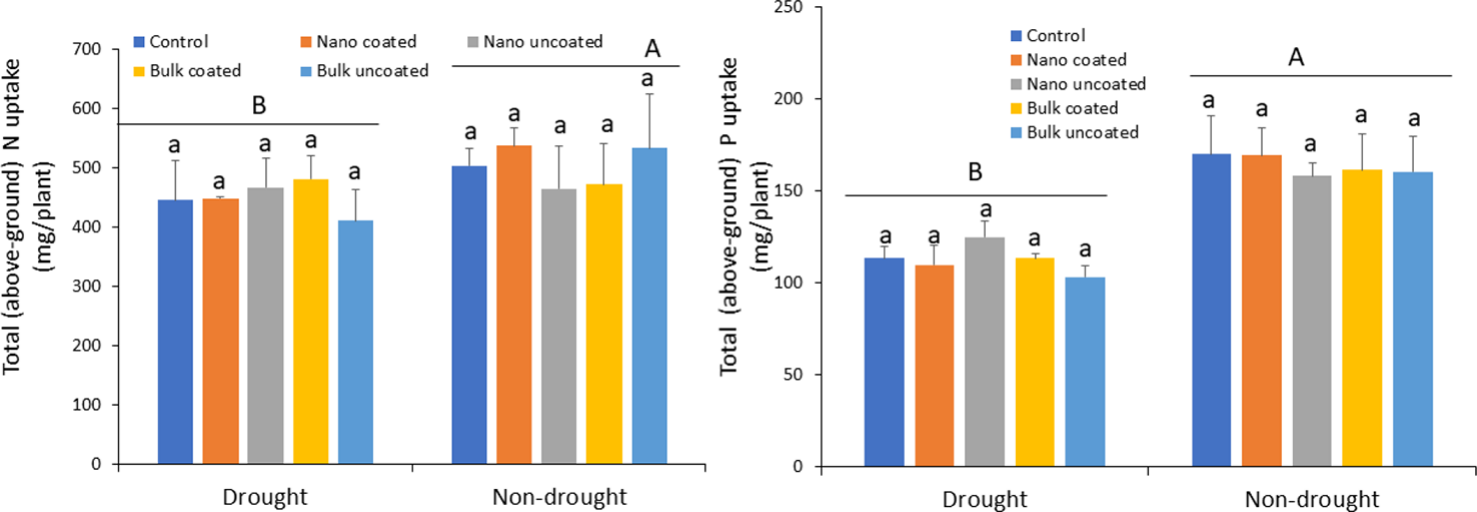
Effect of urea coated with ZnO-nanoparticles (NPs) or bulk ZnO and of separate ZnO-NP or bulk ZnO amendment with urea on above-ground accumulation of nitrogen (left panel) and phosphorus (right panel) in wheat under drought and non-drought growth conditions. Values are means and standard deviations. Different uppercase letters above horizontal lines represent significant difference between the drought and non-drought condition. Different lowercase letters on bars indicate significant differences among the Zn treatments for each growth condition (n = 3). The term uncoated in the legend is with respect to Zn.

Drought strongly reduced N uptake into above-ground (shoot and grain) wheat tissues relative to the non-drought condition by 12–23%, depending on the treatment. However, Zn amendment did not mitigate the effect of drought stress on N uptake, regardless of Zn type, coating or not on urea, and water status of the plants (**Figure 6**; left panel). As with N, drought significantly reduced P uptake into above-ground wheat tissues by 18–37%, relative to the non-drought condition. Notably, Zn amendment also did not mitigate the negative effects of drought on P uptake, regardless of Zn type, whether coated onto urea or not, and the water status of the plants (**Figure 6**; right panel).

### Effects of Zn-Coated Urea on Post-Harvest Soil pH and Residual N, P, and Zn

Plant growth in the soil under drought with and without Zn amendment did not significantly alter soil pH from the pre-planting value of 6.87. Conversely, soil pH was significantly altered by adequate watering as compared to the drought condition; the pH increase under normal watering condition was, however, not significantly influenced by Zn treatment (**Table 2**). Drought caused significantly higher residual N in the soil both as ammonium and nitrate fractions. In both fractions, amendment with Zn did not significantly influence residual N, regardless of water status. However, N existed in the soil more as nitrate than as ammonium in all cases, except for the treatment with separately amended bulk ZnO under both growth conditions (**Table 2**). The residual P level was affected by water status, but not by Zn treatment (**Table 2**). Residual Zn was unaffected by water status of soil but was significantly affected by Zn treatment. Under drought, all Zn treatments increased residual Zn, more so with the bulk ZnO particles. Under non-drought condition, only the bulk oxide treatments significantly increased residual soil Zn (**Table 2**). These effects were similar in each case, regardless of whether urea was coated or not with Zn.

**Table 2:** Post-harvest soil pH and residual bioavailable N, P, and Zn (mg/kg) as affected by drought and Zn treatment.

Trt.	Drought					Non-Drought				
	Control	Nano coated	Nano uncoated	Bulk coated	Bulk uncoated	Control	Nano coated	Nano uncoated	Bulk coated	Bulk uncoated
pH	6.93aB	6.88aB	6.99aB	7.08aB	6.85aB	7.04aA	7.20aA	7.04aA	7.08aA	7.09aA
NH_4_-N	4.86aA	5.99aA	4.45aA	5.16aA	5.13aA	1.99aB	2.04aB	2.08aB	1.89aB	3.86aB
NO_3_-N	6.74aA	8.00aA	5.03aA	6.13aA	4.01aA	2.64aB	3.23aB	3.33aB	2.68aB	3.50aB
P	25.3aA	23.5aA	25.1aA	22.5aA	25.6aA	19.5aB	19.7aB	20.4aB	19.6aB	21.5aB
Zn	0.28cA	0.81bA	0.81bA	1.43aA	1.43aA	0.39cA	0.66bA	0.59cA	1.03abA	1.28aA

Values are means. Different uppercase letters after values represent significant difference between the drought and non-drought condition. Different lowercase letters after values indicate significant differences among the Zn treatments for each growth condition (n = 3). The term uncoated is with respect to Zn.

## Discussion

ZnO-NPs of similar shapes as obtained in this study have previously been observed, and aggregation of ZnO-NPs when suspended in water is also documented (see for e.g., Dimkpa et al., 2012a; Zhang et al., 2018). However, aggregation could also have resulted from drying of the suspension on the TEM grid. Nevertheless, we demonstrate in this study that coating of urea with a low dose of the dry ZnO-NP powder can enhance wheat performance by accelerating phenological development (panicle initiation) and enhancing grain yield and Zn nutrition from a Zn-deficient soil stressed with drought. To the best of our knowledge, this is the first report on the effect of urea coated with ZnO-NPs on crop performance under a challenged growth condition, in this case drought. Coating of bulk fertilizers such as urea can facilitate fertilizer regimes requiring the co-application of macro- and micro- nutrients. However, large-scale field application of micronutrients such as Zn that are required in small amounts by themselves can result in non-uniform distribution of the analytes in the field, resulting in sporadic and unpredictable effects on crop productivity (Santos et al., 2018). Accordingly, physical coating of micronutrient nanoparticles onto bulk fertilizers has been recommended as a viable option to address this problem (Dimkpa and Bindraban, 2018).

Milani et al. (2015) coated urea with ZnO-NPs and bulk ZnO (each at 1.5%) by spraying with a small amount of water that served as a binding agent for the particles, followed by drying. They reported similarly low, < 1%, Zn dissolution from the products in an alkaline calcareous soil, wherein high pH induced by urea affected Zn dissolution due to pH-dependent particle aggregation. However, unlike the alkaline soil, the soil used in the present study was slightly acidic (pH 6.87), and the urea-Zn treatments did not significantly alter pH after plant growth (**Table 2**), likely due to exudation of organic acids that counteracted any urea-induced alkalinity. This suggests that dissolution was the major fate of ZnO for both Zn types, in the coated and uncoated urea systems. This is indicated by the similarly high residual bioavailable Zn levels in the post-harvest soils (**Table 2**), which agrees with a previous study (García-Gómez et al., 2017). Irfan et al. (2018) also coated urea with bulk ZnO particles (2%) using a slurry composed of honey wax, gum arabica (5% each), paraffin wax, or molasses, without and with heating (60°C) under stirring. They reported that Zn solubility after 24 h was greater in the heated system. Compared to the methods of Milani et al. (2012) and Irfan et al. (2018), the procedure described in the present study, though equally facile, was different because vegetable oil and food coloring were used. This could provide different binding properties and Zn solubility, compared to water or the other described binding agents, in addition to adding carbon into a low organic matter containing soil.

Notably, the rate of plant exposure to Zn from urea coated with ZnO-NPs was slightly (6%) lower than with urea with separate ZnO-NP amendment (1.6 *vs*. 1.7 mg/kg). Compared to the bulk ZnO, it was lower by 52–54%, depending on whether coated or not onto urea (see **Table 1**). Importantly, the ZnO-NP-coated urea not only affected panicle initiation time, grain yield and Zn accumulation to the same degree as the uncoated urea with separate ZnO-NP amendment at a slightly higher Zn exposure rate, it also performed well against the bulk ZnO even with significantly lower (52%) Zn rate. Notably, the effect of Zn on panicle initiation at the actual exposure rates was nanoscale-specific under drought stress. We previously reported acceleration of sorghum development by ZnO-NPs, wherein emergence of the flag leaf and grain head was prolonged by drought, but that delay was alleviated by ZnO-NPs (Dimkpa et al., 2019b); bulk ZnO or Zn salt was not co-evaluated in that study. In wheat, Zn salt (1–3 kg/ha; ≈ 0.5–1.5 mg/kg) alone did not affect the number of days to anthesis in plants grown in soil originally containing 0.17 mg/kg Zn. However, the interaction of N and Zn application significantly reduced time to anthesis (Sher et al., 2018). Therefore, the present study in which ZnO-NPs but not bulk ZnO reduced panicle initiation time in the drought-stressed but not in the unstressed plants lends credence to both the nano-specificity and the water status-dependence of these effects. The mechanism surrounding ZnO-NP effects on plant development under drought stress may be related to Zn effecting hormonal induction to regulate root growth for improved adaptation to limited water supply. Indeed, expression of genes related to hormones such as ABA and cytokinins was enhanced by ZnO-NPs in droughted wheat plants, concomitant with modulation of root architecture that helped tolerate the stress (Yang et al., 2018). In addition, soil microbes can facilitate hormonal activity in plants, as microbially-produced hormones can be accessed by plants, contributing to tolerance to drought stress (Defez et al., 2017; Yang et al., 2018). Whereas hormones such as indole acetic acid (IAA) can prolong plant vegetative growth, heavy metals can lower plant IAA levels, as reported in cowpea with impaired vegetative growth in a heavy metal-polluted soil (Dimkpa et al., 2009). Thus, as with some other metals, ZnO-NPs may alter IAA effects in plants by lowering its level, as reported for bacteria (Dimkpa et al., 2012c; Haris and Ahmad, 2017). Hence, the time to initiation of reproductive development in wheat was accelerated in the presence of ZnO-NPs under drought conditions.

The important finding that a lower rate of Zn from ZnO-NPs increased grain yield similarly to higher rates from bulk ZnO highlights the value of nano-scale fertilizers as a tool for reducing the rate of fertilizer input in agriculture, while still maintaining equivalent or even increased yields, compared to bulk-scale fertilizers. Along these lines, Subbaiah et al. (2016) demonstrated that maize yield could be increased to greater extents by ZnO-NPs at doses 60–98% lower than the bulk Zn-sulfate dose. However, unlike our study, the Zn rates evaluated by Subbaiah et al. (2016) were already high, 50–2,000 mg/L, and the application route was foliar, rather than through the soil. In studies involving urea coated with bulk ZnO or Zn-sulfate (0.5–2.0%), wheat grain yield was increased between 5 and 18 or 8 and 22%, compared to the control (Shivay et al., 2008a). The present report of grain yield increase by ZnO-NPs aligns with previous studies with other crops (Dimkpa et al., 2017a; Dimkpa et al., 2017b; Dimkpa et al., 2018b; Dimkpa et al., 2019a; Dimkpa et al., 2019b), while also confirming that coating of Zn onto urea does not lower its fertilizer efficacy due to potential effects on solubility, as indicated by Milani et al. (2012).

Accordingly, the above-ground tissue accumulation of Zn from the nanoscale and bulk ZnO particles was not decreased by coating of the particles onto urea. In fact, Zn accumulation was facilitated by coating at the higher Zn rate in the bulk oxide treatments under drought; and coating was slightly more effective than separate amendment for Zn delivery under non-drought condition in the ZnO-NP system. Moreover, tissue Zn levels were similar between the nanoscale and bulk ZnO treatments, despite lower Zn exposure levels from the nanoscale form. Zn fortification of crops through fertilization has been amply reported (Joy et al., 2015; Dimkpa and Bindraban, 2016). However, limited studies are available on the use of Zn-coated urea for enhancing Zn nutrition of crops; notably, these studies involved bulk ZnO or Zn salt (Shivay et al., 2008a; Shivay et al., 2008b). Therefore, as demonstrated for the first time in the present study for ZnO-NPs, coating of urea with Zn represents a clear strategy for facilitating the delivery of Zn into plants, even at relatively low Zn rates, and irrespective of soil water status. Plausibly, the speciation of Zn in the NP-exposed plants, though not assessed in this study, is likely to be Zn-phosphate, given other reports involving ZnO-NPs and wheat (Dimkpa et al., 2013; Zhang et al., 2018). We observed no effect of Zn treatment on N and P accumulation in the wheat plants. This was somewhat surprising, given previous observations to the contrary (Dimkpa et al., 2018b). For N, such prior observation as it relates to wheat shows that uptake into above-ground plant parts can be increased by as much as 21% with 6 mg Zn/kg soil from ZnO-NPs (same batch used in the current study). However, in the study in question, the N source was ammonium nitrate, rather than urea. Apparently, the effect of Zn as an element in stimulating N uptake can be both N-fertilizer-type and Zn-dose dependent. For example, 4 mg/kg Zn (from Zn-sulfate) had no effect on N uptake, but higher doses of 6 and 8 mg/kg resulted in greater N uptake than in the controls (Abbas et al., 2009). Also, the Zn dose-dependency of N uptake by plants seems species-specific; N uptake was strongly enhanced in soybean when exposure to Zn from ZnO-NPs was at a low rate of 2–3 mg/kg, under drought and non-drought conditions (Dimkpa et al., 2017a; Dimkpa et al., 2019a). With regard to P, Zn amendment is known to inhibit P uptake (Zhao et al., 2000; Abbas et al., 2009; Watts-Williams et al., 2014; Bindraban et al., 2020), due to the formation of insoluble Zn-phosphate complexes. We hypothesize that the temporal and spatial separation of Zn and P applications in the current work could have contributed to the lack of antagonistic effect between these nutrients. Plants are known to utilize significant amounts of P in early root development, which could have lowered the plant-available P concentration prior to Zn-N application.

Collectively, when data on plant development, grain yield and Zn uptake are normalized, there are some interesting hypothetical scenarios based on plant exposure to the same amount (one gram) of Zn. Overall (i.e., under drought and non-drought conditions), these data indicated that there are indeed differences based on particle size. When expressed on the basis of per gram of Zn, panicle initiation time was accelerated with ZnO-NPs than with the bulk ZnO, and coating had no effect in each case. However, this effect was only found in the drought condition; whereas in the non-drought condition only uncoated bulk ZnO facilitated panicle initiation. For grain yield, a nano-specific effect was found in both growth conditions, and there was no effect of coating. For Zn uptake, size effect of ZnO-NPs was strongly demonstrated, as was the effect of coating of ZnO, irrespective of growth condition. Drought enhances plant root exudation for stress adaptation (Karlowsky et al., 2018), which likely would affect the rate of particle dissolution, depending on specific metabolites in the exudate (Martineau et al., 2014; Yang et al., 2018). Hence, Zn was sufficiently bioavailable from ZnO-NPs under drought, even at a lower exposure rate than bulk ZnO. Nevertheless, additional studies to optimize coating efficiency to deliver same Zn rate in urea as the separate Zn addition for both ZnO-NPs and bulk ZnO could provide important insight on the significance of these assumptions.

The significantly higher residual soil N in the drought-exposed plants relative to the normal watering regime may reflect the fact that biomass growth, and thus, N assimilation, was lower in the droughted plants (data not shown). It was observed that N generally existed in the soil more as nitrate than as ammonium, except in the case of uncoated bulk ZnO in both growth conditions. The significance of bulk ZnO altering the dynamics of N types in the soil is currently unclear. However, the presence in soil of more nitrate than ammonium may reflect the transformation dynamics of different N-fertilizer types. Moreover, soil pH and specific plant uptake requirements for either ammonium or nitrate would also play a role in the ratios of the residual nutrients. In this case, the soil being more acidic than alkaline could have induced a preference for ammonium uptake (Maathuis, 2009), resulting in more nitrate being left as residual N in the soil. In our prior study with wheat (Dimkpa et al., 2018b), ZnO-NPs did not reduce residual soil N levels, similar to the current finding. As with N, Zn treatment with either nanoscale or bulk ZnO did not affect residual soil P levels, similar to the previous finding with ZnO-NPs (Dimkpa et al., 2018b). However, these are contrary to ZnO-NPs increasing residual soil P in sorghum (Dimkpa et al., 2017b). In terms of residual Zn, the difference in the levels of Zn exposure between the ZnO-NP and the bulk ZnO soils directly reflects the exposure rates, although merely doubling the obtained mean values for the nanoscale form would suggest higher soluble Zn from the NPs than from the bulk product.

As noted, the soil used in the study has a low organic matter content. The role of the carbon that would be added to the soil by the trace amounts of oil and food coloring used for coating in enhancing soil organic matter is unknown at this time. However, it should be noted that all the urea, with and without ZnO, also was coated with the oil, which makes any effect of coating uniform among the treatments.

The findings reported herein indicate that Zn in nanoparticle form can accelerate wheat phenological development, reproductive yield and Zn nutrition under drought stress, as previously reported for sorghum. A broader implication of this study is that a lower Zn rate from ZnO-NPs may suffice for enhancing crop productivity under drought stress, compared to higher input from bulk ZnO. This clearly demonstrates one of the goals of nano-enabled agriculture, which is to reduce the rate of nutrient inputs into the biosphere without a yield penalty. Coating urea or other N-fertilizers with nano-scale micronutrients such as Zn may increase crop yield; facilitate the use of nanoscale micronutrients in field applications; eliminate the problem of segregation of the smaller and larger nutrient particles in bulk fertilizer blends; and facilitate one-time Zn-urea application. However, coating may not necessarily influence yield to a greater extent than separate Zn and urea applications, as observed in this study. This is especially true as the Zn particles will eventually dissolve from the urea surface and undergo independent transformation to ions or larger aggregates, similar to separately-applied ZnO particles. That being said, it is very likely that making improvements to the urea coating process to increase the coating efficiency of ZnO-NPs will further improve outcomes on crop performance and Zn acquisition.

## Data Availability Statement

The raw data supporting the conclusions of this article will be made available by the authors, without undue reservation, to any qualified researcher.

## Author Contributions

CD: acquired research funding, conceived research, designed experiments, conducted experiments and wrote manuscript. JA: conducted experiments and revised manuscript. JF: conducted experiments, evaluated data and revised manuscript. US: designed experiments evaluated data, and revised manuscript; PB: conceived research and revised manuscript. WE: acquired research funding, conceived research and revised manuscript. JG-T: acquired research funding, conceived research and revised manuscript. JW: acquired research funding, conceived research and revised manuscript.

## Funding

This study was funded by United States Agency for International Development (USAID)'s Feed the Future Soil Fertility Technology Adoption, Policy Reform and Knowledge Management Project (Cooperative Agreement Number AIDBFS-IO-15-00001), and by a U.S. Department of Agriculture (USDA) Agriculture and Food Research Initiative (AFRI) Nanotechnology for Agriculture and Food Systems Grant (2016-67021-24985).

## Conflict of Interest

The authors declare that the research was conducted in the absence of any commercial or financial relationships that could be construed as a potential conflict of interest.
